# Needs for a gender‐based perimarital couples’ counselling services in Iran

**DOI:** 10.1002/nop2.692

**Published:** 2020-11-20

**Authors:** Masoumeh Simbar, Fatemeh Rahmanian, Soheila Nazarpour, Ali Ramezankhani, Farid Zayeri

**Affiliations:** ^1^ Midwifery and Reproductive Health Research Center School of Nursing and Midwifery Shahid Beheshti University of Medical Sciences Tehran Iran; ^2^ Department of Midwifery School of Nursing and Midwifery Shiraz University of Medical Sciences Shiraz Iran; ^3^ Department of Midwifery Chalous Branch Islamic Azad University Chalous Iran; ^4^ Department of Public Health Faculty of Health Shahid Beheshti University of Medical Sciences Tehran Iran; ^5^ Department of Biostatistics Faculty of Paramedicine Shahid Beheshti University of Medical Sciences Tehran Iran

**Keywords:** gender‐sensitive, needs, nurses, nursing, perimarital counselling, reproductive health, services

## Abstract

**Aim:**

To assess needs for a gender‐sensitive perimarital counselling services in Iran.

**Design:**

A descriptive cross‐sectional study.

**Methods:**

This was a study on all 236 premarital counselling providers in 37 health centres in Shiraz‐Iran. The tools for data collection included the following: (a) a demographic information questionnaire and; (b) a valid and reliable Needs Assessment questionnaire for Gender‐Sensitive Perimarital Counseling Services (GSPCS) in 3 sections of needs for process, structure and policy making of the perimarital counselling services. Data were analysed using SPSS 21.

**Results:**

All health providers with average working experience of 8.63 (*SD* 5.35) years participated in the study. Results demonstrated highest scores for needs related to facilities as the structure of the services (90.09 *SD* 13.70 per cent) and community empowerment (89.50 *SD* 16.67 per cent) as the necessary policy for the gender‐sensitive services. We concluded that providing gender‐sensitive perimarital counselling healthcare services needs to reform the process, structure and policies of the services.

## 1 INTRODUCTION

Providing gender‐sensitive health services especially reproductive services is emphasized in recent decades (Cochrane & Rao, 2019; Heckert et al., 2019). Sensitivity for gender issues in health care means that health authorities have the knowledge and are able to perceive existing gender differences and to integrate these into their decision‐making and actions (Celik et al., 2011).

## 2 BACKGROUND

Perimarital counselling helps couples to initiate their common marital life with the necessary information (Salarvand et al., 2011) and prevents dissatisfaction and failure in marital life and so it is considered as an educational preventive approach (Khaleghinejad et al., 2009). Couples need appropriate and accurate information about different aspects of reproductive health such as STIs/HIV/AIDS prevention and contraceptive use which are from important issues in marital life. Therefore, perimarital health counselling services are important as these help couples to initiate a healthy sexual relations and life (Ramazani et al., 2013). Besides, perimarital counselling about incorrect gender norms improves couples' understanding and relations from beginning of their marital life. (WHO, 2010).

Perimarital counselling in primary healthcare services needs high attention in middle‐eastern countries as they are facing with polarization of their culture into traditional and modern cultures about sexual behaviours (Women, 2019). While some people choose premarital sexual activity, some others consider even talking about sex as a taboo (Ussher et al., 2017). Hymen examination as the “Virginity testing” is still important for traditional Iranian families because they think intact hymen means no sexual relationship before marriage (Simbar et al., 2015), while this custom results in non‐vaginal sexual relationships such as anal and oral relationship and hymenoplasty before marriage (Robatjazi et al., 2016a, 2016b). Studies in Iran show majority of couples consider sexual and reproductive health affaires as a feminine issue and reproductive health system such as perinatal services are mostly defined and provided for women not men (Eskandari et al., 2017; Simbar et al., 2010). In a study by United Nation Population Fund (UNFPA), patriarchy, early marriage and son preference are the most effective barriers of women's equality and equity in Asia especially in Middle East countries (UNFPA, 2005). Besides, talking about sex with adolescents is a taboo in many Muslim countries (UNFPA, 2005) and therefore youth may not be prepared to protect themselves against STIs/HIV/AIDS and unwanted pregnancy until marriage (Banaei et al., 2019) and even after marriage (Khalesi et al., 2017). Besides, information about prevalence of STIs among non‐married youth in Islamic countries including Iran, where non‐marital sex and homosexuality are prohibited by religion and culture, is notably limited. Some of the STIs preventive strategies that are advocated and used in non‐Islamic countries are not acceptable in Islamic countries. For instance, the concept of "Safe Sex" to prevent STIin non‐Islamic countries basically promotes the use of condoms for non‐marital sexual relations, considered in Islamic countries a way of promoting out of marriage sex which is absolutely prohibited in Islam (Madani, 2006). Therefore, the effective cultural factors and the gender‐based discriminations in any community should be known before any interventional programmes to promote family and marital health (Jacob, 2017). Otherwise, to plan an effective reproductive health educational programme, extensive anthropological and sociological needs assessments are necessary to know about gender roles and stereotypes in a sexual and marital life in the family and social framework and their specific physiological needs (Faludi & Rada, 2019).

Premarital counselling is integrated in PHC services in the recent decades in Iran. It began with educating couples about preventing unplanned pregnancy and STIs/AIDS prevention. Its certification is a requirement for conventional marriage (Khalesi et al., 2016; Mehrolhassani et al., 2018). This service was criticized because of inappropriate time, duration and content, for instance, the service were provided when couples are involved with heavy task related to their marriage ceremony (Khalesi et al., 2017). Now, the educational content develops to provide sexual health and couples’ communication skills (Dabiri et al., 2019).

However, in our knowledge there is no study to understand gender‐based needs of couples for perimarital counselling. Therefore, this study aims to assess needs for a gender‐sensitive perimarital counselling services based on the perspectives of experienced providers of perimarital counselling with a valid and reliable questionnaire.

## 3 METHODS

### 3.1 Design

This was a descriptive cross‐sectional study in Shiraz‐Iran 2018.

### 3.2 Participants

All 236 providers of couples’ premarital counselling, with at least 2 years work experience including physicians, midwives and health educators from all 37 health centres in Shiraz‐Iran, participated in the study. The sample size was calculated using a pilot study to calculate sample size based on the following formula:

$$n=\frac{Z^{2}\alpha /2.S^{2}}{d^{2}}$$

$$d=k\bar{X^{\prime }}$$

*S*: 9.8

*Z*α/2 = 1.96 (For 95% Confidence Interval)

*K* = 0.02 (0.01–0.05)

*X*' = 62.66 (Range: 0–74) − (Calculated from a pilot study)

### 3.3 Tools for data collection

Two questionnaires were used including (a) a demographic information questionnaire and (b) a valid and reliable questionnaire to assess needs of Gender‐Sensitive Perimarital Counseling services (GSPCS) (Rahmanian et al., 2014). This was a self‐completed questionnaire which could be filled up in average time duration of 20 min.

GSPCS included 37 questions in 8 subscales including.

(a) needs for care (3 items); (b) educational needs (5 items), (c) needs for human resources (8 items); (d) needs in facilities (4 items); (e) needs for management (4 items); (f) needs for intersectional cooperation (4 items); (g) needs for supportive policies (6 items); and (h) needs for community empowerment (3 items).

Each item of the questionnaire was scored by a 3‐point rating scale from “not necessary,” “somewhat necessary” to “completely necessary” which scores from 0 to 2. The scores of items were summed up for each sub scale and total scale and then converted to per cent (0–100).

GSPCS was developed by authors of the present study through a qualitative study by content analysis approach and an extensive literature review (inductive‐deductive method for the tool development) in Persian language. Then Content validity index and content validity ratio of the GSPCS were calculated 0.99 and 0.95, respectively. Reliability of GSPCS is also confirmed by alpha Cronbach 0.96 for internal consistency and intra‐cluster correlation coefficient 0.804 of test retest for stability. Data were analysed using SPSS 21.

### 3.4 Procedure of the study

Two hundred and thirty‐six counselling providers with at least two years’ experience in premarital counselling from all 37 health centres in Shiraz agreed to participate completed the questionnaires. The questionnaires were self‐completion forms. The questionnaires were completed by responders anonymously. The responders were ensured about the confidentiality of their personal information, by providing explanation about anonymous responses and keeping and using information just for the study's objectives. All participants contributed to the research after giving the informed consent. All 236 counselling providers accept to complete the paper questionnaires, and all completed the questionnaires completely and so no drops were happened. The questionnaires were collected, and the data were analysed.

### 3.5 Data analysis

Data were analysed using SPSS 21 and descriptive analytical methods such as calculating frequency and percentage and sorting results for prioritizing of the needs.

### 3.6 Ethics

The study was approved by the Ethical committee of Shahid Beheshti University of Medical Sciences. All participants of the study gave a written consent for their participation in the study.

## 4 RESULTS

Two hundred and thirty‐six premarital counselling provides with average age of 32.90 (*SD* 6.48) years and with working experience duration of 8.63 (*SD* 5.35) years participated in the study. Majority of the counselling providers were female and midwife. Table 1 shows the participants’ demographic characteristics. Results demonstrated highest needs for facilities and community empowerment. Table 2 shows mean and standards deviation of 8 dimensions in 3 sections of perimarital counselling services. Table 3 shows high priorities (items with highest scores) in each dimension of premarital counselling services.

**TABLE 1:** Demographic characteristics of the perimarital counselling services providers in Shiraz 2016 (*N* = 236)

Characteristics	Category	Number	Per cent
Gender	Female	194	82.2
	Male	42	17.8
Age	20–30	110	46.6
	31–40	97	41.1
	>40	29	12.3
Education	Midwife (Bachelor)	140	59.3
	Health educator (Bachelor)	42	18.6
	Midwife (Master)	19	8.1
	Physicians	17	7.2
	Midwife (Graduate Diploma)	16	6.8
Job experience (Years)	2–10	165	69.9
	11–20	66	28.0
	>20	5	2.1

**TABLE 2:** Mean and standard deviation scores for needs of gender‐sensitive premarital counselling services in 3 sections and 8 dimensions of the services

Sections	Dimensions	Mean ± *SD* (score 0–100)	Mean ± *SD* (score 0–100)
Process	Care	89.40 ± 15.62	89.22 ± 13.35
	Education	89.11 ± 15.20	
Structure of the Services	Facilities	90.09 ± 13.70	87.72 ± 12.68
	Human resources	87.31 ± 14.52	
	Management	86.17 ± 16.79	
Gender‐sensitive policies	Community empowerment	89.50 ± 16.67	86.29 ± 12.38
	Supportive policies	88.38 ± 14.01	
	Intersectional cooperation	80.72 ± 18.09	

**TABLE 3:** High priority needs in each dimension of premarital counselling services

Dimensions Of need	Priorities	Score		Frequency (per cent)		
		Mean (0–2)	*SD*	Not necessary 0	Somewhatnecessary 1	Completely necessary 2
Care	Postmarriage continuous care and counselling for sexual reproductive health problems	1.80	0. 4	0.4	18.6	80.0
	Providing possibilities for examination and screening of male's and female's reproductive system disorders	1.80	0.42	0.8	18.2	81.0
Education	Education about hymen and correcting of myth about that	1.81	0.41	0.8	16.9	82.3
	Couple's education about interpersonal skills based on reproductive rights	1.80	0.42	0.8	18.2	81.0
Facilities	Providing facilities for diagnosis, treatment and follow‐up of STIs for men	1.86	0.33	13.0	13.0	74.0
	Professional counselling based on clients’ age	1.80	0.40	0	19.9	80.1
Human resources	Employing trained personnel for sexual health counselling	1.86	0.37	0.8	12.1	87.1
	Educating personnel on skills for couples’ counselling	1.80	0.42	0.8	18.0	71.2
Management	Management of comprehensive services for premarital counselling and female adolescents’ health	1.77	0.41	0	22.5	77.5
	Providing a gender‐based educational content and counselling approach	1.74	0.46	1.3	23.3	75.4
Community empowerment	providing opportunities for education before choosing a spouse at the community level	1.87	0.35	0.8	10.9	87.3
	providing counselling without discrimination in relation to sexual desire in society	1.77	0.41	0	22.2	77.8
Intersectional cooperation	Teaching essential points a spouse in school health	1.66	0.48	0.8	31.0	68.2
	Planning for comprehensive sexual health education in health services	1.64	0.55	3.3	31.4	65.3
Supportive Policies	Planning a STIs risk assessment programme in counselling process	1.83	0.38	0.4	15.5	84.1
	Planning for individuals’ education on negotiation skills for sexual health	1.79	0.43	1.3	17.6	81.1

## 5 DISCUSSION

This was the first study to assess needs for providing a gender‐based perimarital counselling service in Iran. Findings of present study showed the highest priorities for reforms in the structure especially facilities of the pre‐marriage services, and policy making by community empowerment through providing opportunities for the education before choosing a spouse at the community level.

The most important care was demonstrated to be “Postmarriage continuous care and counselling for sexual reproductive health problems” and then “Providing possibilities for examination and screening of male's and female's reproductive system disorders.” This study demonstrated a lack of continuity in marriage counselling services in the present health system. Although the present counselling services provide a good opportunity to increase couples’ knowledge about sexual health and rights but it is not responder to their postmarriage problems, questions and needs such as about their reproductive system (Bostani Khalesi & Simbar, 2017; Bostani Khalesi et al., 2015). In a study in eight countries, Hardy et al. found that an empowering environment that includes specialized health services for young women and men and support services can reduce sexual abuse, violence, sexual dissatisfaction and communication problems (Hardee et al., 1999).

Findings showed two priorities in the educational needs including “Education about hymen and correcting of myth about that” and “Couple's education about interpersonal skills based on reproductive rights.” Lack of knowledge about anatomic condition of hymen, the myth of “intact hymen means chastity” and the rejection of girls from family for hymen injuries following prohibited sexual relations are from important issue in relation to inequality imposed on girls (Robatjazi et al., 2016a, 2016b; Simbar et al., 2015). Communication skills between men and women are recognized as the most important factors in promoting sexual health, preventing the spread of sexually transmitted diseases (Allen et al., 2007). In our country, taboo of talking about sexual relations and sexual health education starts from schools; so pre‐marriage counselling makes appropriate opportunity for correcting thoughts and taboos (Bostani khalesi et al., 2015; Khalesi et al., 2016).

Results demonstrated highest scores for the service structural reform through improving facilities including “Providing facilities for diagnosis, treatment and follow‐up of STIs for men” and “Professional counselling based on clients’ age.” Reform in all areas of reproductive health services is important as men are usually deprived of the services because of the traditional focus of clinics on serving women and children (Narges Eskandari et al., 2016; Eskandari et al., 2016). Therefore, providing services in appropriate time and place for men are essential (Simbar et al., 2010). Although establishment of such services is considered to improve men's reproductive health, it is very costly and inoperative. Successful experiences demonstrated that changing clinics’ working hours in the afternoon and holidays, hiring male personnel and creating the right physical environment for men is possible to improve these services in the existing system (Simbar et al., 2011; Simbar et al., 2010). The gender of provider is an important component of gender‐sensitive services because cultural and social barriers impede men from referring to reproductive health services that all employees of which are women (Alimoradi & Simbar, 2014). Besides, female health providers are also not interested to provide information on sex issues to male clients (Bostani khalesi et al., 2015; Verdonk et al., 2009). Understanding the needs of men and responding to these needs has contributed to their health promotion; and the service providers must be able to address these needs including: avoiding high‐risk sexual relationships, preventing unwanted pregnancy, choosing the number and date of birth of children, fathers’ understanding for helping pregnant wife, protecting themselves and their spouses against STIs, screening and treating STIs, treating fertility problems, increasing self‐esteem, awareness and skills for avoiding violence and forced sexual relations (Alimoradi, 2017).

The priorities in the dimension of necessary human resources for gender‐sensitive premarital counselling services were “Employing trained personnel for sexual health counselling” and “Educating personnel on skills for couples’ counselling.” The most important reason for failure of providers in sexual health counselling is lack of education in the field of sexual counselling. Sexual issues are from private area of individuals and counsellor should be trained and skilful to communicate with clients effectively to talk about sexual health. However, many sexual health providers are even not willing to consult and discuss in this area (Bostani khalesi et al., 2015).

Findings showed the highest scores for the needs for “Management of comprehensive services for premarital counselling and female adolescents’ health” and “providing a gender‐based educational content and counselling approach,” respectively. Female adolescents are vulnerable to reproductive problems and need high‐quality counselling services, because of low experience, female adolescents are not able to reject high‐risk relationships (Alimoradi et al., 2019; Shahhosseini et al., 2013). It is very difficult for them to discuss and convince spouses about the use of condoms because their husbands are usually older than them and even have sexual experiences. Moreover, the juvenile genital system is more vulnerable to infection, and this factor along with the lack of reproductive health information increases the risk of sexually transmitted diseases and AIDS among female adolescent (UNICEF, 2005). Generally, early marriage leads to early motherhood and adolescence high‐risk pregnancies (ICRW, 2005).

Concerning community empowerment in the promotion of pre‐marriage counselling services, "providing opportunities for education before choosing a spouse at the community level," and then "providing counselling without discrimination in relation to sexual desire in society," showed highest scores. Taboo of talking about reproductive issues cause to reduce effective communication between health system, schools and even parents with their children, while supportive family and youth friendly services (Shahhosseini et al., 2012) which provide these educations before choosing a spouse have had successful results (Bahrami et al., 2013).

Finding demonstrated the two top priorities in the intersectional cooperation dimension of the policy section including "Teaching essential points before choosing a spouse in school health" and “Planning for comprehensible sexual health education in health services." Since reproductive behaviours and marriage are influenced by social, cultural and individual conditions, successful interventions for premarital counselling should be planned based on adolescents’ perspectives and experiences on a specific social context (Chandra‐Mouli et al., 2019; El Kazdouh et al., 2019). Schools are from the best centres where sexual health can be promote among young people (Bahrami et al., 2013; Shahhosseini et al., 2016).

In the section of supportive policies, the needs for “Planning a STIs risk assessment programme in counselling process " and “Planning for individuals’ education on negotiation skills for sexual health” took the highest scores from the point of view of the participants. In the cultures that men and women suffer from sexually transmitted diseases that leads to violence, isolation and social stigmatization for women, a risk assessment system should be designed for overcoming the barriers of detected high‐risk behaviours and using experience of other countries (Khalesi et al., 2016; Lichtenstein, 2003; Medley et al., 2004). A review also showed that health plans that consider couples as the target group were far more successful than programmes that define each male and female as a target group (Tokhi et al., 2018). Also, interventions can have better results if consider people's reproductive health rights (Population Council, 2005).

### 5.1 Limitations of the study

The taboo for discussing about sexual and gender issues was the main limitation of this study, which was partially controlled by keeping confidentiality of the names of individuals, explaining about goals and intimate encounters with the participants. Healthcare providers who recruited as the participants of the present study live in our traditional society and they may follow the same myth and traditions of the community. However, they receive a comprehensive sexual education in academic courses but gender‐based effective factors on couples’ health during marital life are not considered in the courses. Besides, it is shown that talking about sex topic is hard for counselling, especially when providing sexual counselling for clients of opposite sex is necessary (Pourmohsen et al., 2018). It seems the healthcare providers beliefs should also be corrected first of all.

## 6 CONCLUSION

Perimarital counselling services can be improved by paying attention to gender‐sensitive needs. The top priorities for reforming the present perimarital counselling are as follows: community empowerment through providing opportunities for education before choosing a spouse; the advocating policies for planning a STIs risk assessment programme in counselling process; improving facilities for counselling preventing, diagnosis, treatment and follow‐up of men's sexually transmitted diseases; employing trained personnel for sexual health counselling; continuity of counselling and care to postmarriage sexual and reproductive health problems; and education about hymen and correcting of the myth.

Otherwise, the findings of this study suggest that cultural structure need to reform at the community level to correct some of misconceptions such as “considering the hymen examination as a virginity test" but also structural reforms are necessary in the health management section such as the employment of trained male personnel to provide premarital sexual reproductive health counselling, and providing space and facilities for providing sexual health counselling services for both women and men. In addition, appropriate educational design and content for counselling at clinical level are essential with considering cultural factors related to gender affecting the couple's sexual reproductive health and relationship after marriage.

### 6.1 Implications

World Health Organization emphasizes on improving quality of reproductive health services including premarital counselling services by providing gender‐sensitive services. Improving quality of services in all 3 dimensions of healthcare services including structure, procedure and outcome is essential. The present study assessed the needs for providing a gender‐sensitive premarital care services in all 3 dimensions using a standard questionnaire and showed the gaps and needs of the system to reform. The process of the present study and its needs assessment tool can be applicable in similar services for needs assessment and showing the gaps of the system which needs intervention and reform for providing quality gender‐sensitive services. The research shows gender‐based educational planning are necessary to integrate in present programmes to improve quality of perimarital care and counselling programmes. Therefore, further research is suggested to find other culturally gender‐based barriers and facilitators to improve quality of care in other aspects of sexual reproductive health such as in the area of family planning and adolescents health.

## CONFLICTS OF INTEREST

The authors declare they have no conflict of interest.

## AUTHOR CONTRIBUTIONS

All authors: substantial contribution to and agreement on the final version of the manuscript.

## Data Availability

The datasets used and/or analysed during the current study are available from the corresponding author on reasonable request.
